# Dupuytren’s disease and the risk of malignant neoplasms

**DOI:** 10.1186/1897-4287-12-6

**Published:** 2014-03-06

**Authors:** Andrzej Żyluk, Katarzyna Paszkowska-Szczur, Satish Gupta, Rodney J Scott, Jan Lubiński, Tadeusz Dębniak

**Affiliations:** 1 Department of General and Hand Surgery, Pomeranian Medical University, Szczecin, Poland; 2 Pomeranian Medical University, Department of Genetics and Pathomorphology, Szczecin, Poland; 3 Discipline of Medical Genetics, Faculty of Health, University of Newcastle and Hunter Medical Research Institute, Newcastle, NSW, Australia

**Keywords:** Dupuytren’s disease, Cancer risk, DD

## Abstract

The object of this study was the investigation of the risk of occurrence of malignant neoplasms in 508 patients with Dupuytren’s disease (DD) and in 2157 of their 1st degree relatives. In the first stage of the study, we evaluated the tumour spectrum as well as the age of the patient at diagnosis of cancers in DD families along with the observed and expected frequencies of malignancies. In the second stage of the study, we examined the distribution of 20 common mutations/polymorphisms in 12 known cancer susceptibility genes among DD patients and 508 matched healthy controls. No such study has been published to date. Results. No significant differences were noted between malignancies diagnosed among members of DD families and the general population. Molecular examination of 20 mutations/polymorphisms in 12 cancer susceptibility genes in Dupuytren’s patients and controls showed a statistically significant association of one mutation with Dupuytren disease: D312M in XPD (OR = 1.75, p = 0.004). We observed a tendency toward changed frequencies of occurrence of central nervous system tumors, laryngeal cancer and non-melanoma skin cancers in DD families. The results of our study indicate a lack of a strong association between Dupuytren disease and familial cancer risk.

## Background

Dupuytren’s disease (DD) is classified as a benign superficial fibromatosis, in which excessive proliferation of myofibroblasts result in the formation of nodules and chords occurs, followed by the development of finger contractures. Prevalence of DD is geographical and racially related, with the greatest morbidity in Northern Europe and it more commonly affects Caucasians than other ethnicities [1]. Dysregulation of specific genes may have an effect on fibroblast growth characteristics in palmar aponeurosis leading to their progressive differentiation into myofibroblasts and an over-production of type III collagen [2].

There are several similarities between DD and malignant neoplasms, suggesting a possible association between these pathologies. Similar to cancer cells, DD fibroblasts are characterised by infiltrative growth, proliferation, lack of apoptosis and a tendency towards recurrence. Molecular studies showed that DD fibroblasts and solid tumor cells present a similar quality and quantity of chromosomal aberrations (translocations or trisomies) and the capability to bind monoclonal antibodies derived form human sarcomata [3,4]. Immunochemical studies revealed decreased expression of Rb and p53 tumor suppressor genes within these fibroblasts, which is a characteristic feature of many cancers [5,6]. We have previously reported a possible association between common variants of *DHDH* and the familial occurrence of DD [7]. It was reported that down-regulation of *ALDH2* and *DHDH* might be associated with a higher risk of gastrointestinal tract cancers in alcohol consumers [8,9].

Modest literature data on the possible association between DD and malignant neoplasms prompted us to explore this potential association. We have investigated the risk of occurrence of malignancies at a different site of origin in a series of 505 Dupuytren patients and 2157 their 1st degree relatives. We evaluated the tumour spectrum, patients’ age of cancer diagnosis in DD families and the observed (actual) and expected frequencies of malignancies. Mutations or polymorphisms of many cancer susceptibility genes, such as BRCA1, CDKN2A, XPD, VDR, lead to tumour development. To evaluate the possible link between Dupuytren’s disease and malignant neoplasms, we examined additionally the distribution of 20 mutations/polymorphisms in 12 known cancer susceptibility genes among patients and 505 matched healthy controls. To our knowledge, no such investigations have been performed to date.

## Materials and methods

### Patients and controls

Over a period of five years (form 2008 to 2012), 508 patients with Dupuytren’s disease, 410 men (81%) and 98 women (19%) with a mean age of 57 years (range 38–86) were recruited. One hundred and twelve patients underwent surgery for DD before 2008. They were identified in the institutional Dupuytren’s disease register and were invited to participate in the study by mail. Three hundred and ninety six patients were recruited during their stay in the hospital for operation. These patients were referred to our institution for Dupuytren’s surgery over the period of the study 2008–2012. The approval of the Bioethical Council of the local Medical University was obtained and informed consent was obtained from all subjects before enrolment. During an interview the goals of the study were explained, genetic counselling was given and a blood sample was taken for DNA analysis. A detailed family history and the duration of DD were recorded. The total number of 1st degree relatives (including patients) was 2662 (1224 females and 1438 males).

The control group comprised 508 healthy adults, 410 men and 98 women with a mean age of 57 years (range 38–86) who were age matched (+/−2 years) with the DD patients. The healthy adults were assessed as having a negative family history for cancer after answering a questionnaire about their family medical history, which was part of a population-based study of the 1.5 million residents of West Pomerania province to identify familial aggregations of malignancies. A blood sample was taken for DNA analysis from all controls.

### Methods

In the first stage of the study, we compared frequencies of malignant tumour occurrence and patient age at diagnosis in DD families against those of the general Polish population. We evaluated malignancies affecting probands and their 1st degree relatives.

For statistical analyses, the Chi-Square test, U-test and odds ratio were utilised. Additional analyses performed in DD families included the comparison of observed frequencies (OF) with expected frequencies (EF) and the relative risk (RR) of occurrence of malignancies at different sites of origin. OF and EF were calculated by evaluation of the total number of family members and affected cases in different age groups (range of 5 years) in DD families in comparison to age-specific incidence rates in different age groups (range of 5 years) per 100 000 people. These calculations were done by site with individuals registered in the general population of Poland [10]. Bonferroni correction was used for multiple testing.

In the second stage of the study, we compared the prevalence of common alterations in DNA reported in literature with cancer susceptibility alterations among

– DD patients,

– healthy controls, and

– general authors’ native population

(data obtained from already published reports). We genotyped founder BRCA1 mutations (5382insC, C61G, 4153delA), common variants of XPD (D312M, K751Q, R156R), XPC (A499V), XPF (c.207 + 11G > A), NOD2 (3020insC), CDKN2A (A148T), CHEK2 (CHIVS2, I157T), VDR (M1T), p53 (P72R), ATM (E1978X), MC1R (R160W, R151C, R163Q), MTHFR (A222V), and rs67 (rs6983267).

All SNPs were analysed by real-time PCR, using a LightCycler480 from Roche. The analyses were performed using the TaqMan(R) genotyping assay, which consists of sequence specific primers and oligonucleotide fluorescent labelled probes, enabling amplification of the examined fragments and further allele discrimination. Randomly selected probes were sequenced to confirm the results of real-time PCR.

Power calculations were done *a posteriori* based on the observed vs expected frequencies, the number of individuals analysed and the threshold p-value (after Bonferroni correction for multiple testing). The significance threshold for the p-value was 0.05/20 for analysis of the genetic markers, and in the case of analysis via the cancer site, the threshold was 0.05/24 for women and 0.05/22 for men (0.05 divided by the amount of hypotheses tested for the same group).

The statistical power was calculated using “pwr” package of R-version 2.15.0). The alpha (α) value for men was 0.0023 (0.05/22) and for women was 0.0021 (0.05/24).

## Results

Evaluation of the mean patients’ age at the diagnosis performed in our families for cancers affecting at least 5 individuals revealed no significant differences between malignancies diagnosed among members of DD families and the general population (Table 1).

**Table 1:** Proportion and age at diagnosis of malignancies of different site of origin in Dupuytren’s disease families and polish population

**Tumor site**	**Males from Dupuytren’s disease families**		**Population (males)#**		**Females from Dupuytren’s disease families**		**Population (females)#**	
	**Frequency**	**Mean age at diagnosis (yrs)**	**Frequency**	**Mean age at diagnosis (yrs)**	**Frequency**	**Mean age at diagnosis (yrs)**	**Frequency**	**Mean age at diagnosis (yrs)**
	**(%)**		**(%)**		**(%)**		**(%)**	
Breast	0	ND^	0,2	64,1	28,8	56,95	22,4	60,2
Lungs	21,8	65,38	21,1	65,9	4,32	63,33	8,6	65,1
Colorectal	13,3	70,21	12,4	67,2	7,91	65,64	10,1	68,3
Stomach	10,3	63,88	4,9	66,4	3,6	65,8	2,7	68,6
Prostate	13,9	68,91	13,2	70,1	-	-	-	-
Kidney	3,03	65,8	4,1	63	4,32	63,67	2,8	65,2
Larynx	6,67	64,64	2,7	62,4	0	ND^	0,4	62,2
Skin	3,64	67,5	6,8	69,3	1,44	ND^	7,5	70,2
Leukaemia	3,03	57,38	2,2	56,9	2,88	ND^	1,9	62
CNS	7,88	56,38	2,4	55,3	7,91	62,45	2,3	59,3
Pancreas	2,42	ND^	2,3	64,7	5,04	70,43	2,3	69,2
Uterus	-	-	-	-	4,32	54,5	7,3	63,6
FGT*	-	-	-	-	12,2	57,94	17,8	68,8

#-general Polish population, according to data published by National Cancer Registry [10].

^ND-not done-mean age was calculated for malignancies affecting at least 5 individuals.

FGT-female genital tract- site of origin could not be determined more precisely.

Evaluation of tumor spectrum revealed moderate differences between DD families and the general population:

– laryngeal cancer constituted larger proportion of cancers affecting males from DD families (6.7%) when compared to males from general population (2,7%);

– central nervous system tumors were overrepresented among malignancies affecting both males from DD families (7.9% vs 2.4% in general population) and females (7.9% vs 2.3%);

– non-melanoma skin cancers were under-represented in DD families (3,6% of DD male neoplasms vs 6,8% in the general population and 1,4% of DD female malignancies vs 7,5% in the Polish population).

We observed 288 cancers among 2662 members of 508 DD families. In comparison to the expected frequency (266 tumours), the difference was not significant (p = 0.35), neither for males (p = 0.13) nor females (p = 0.90).

A statistical comparison of the observed and expected frequencies of neoplasms revealed no statistically significant differences between DD families and the general population. There was a non-significant over-representation for central nervous system tumors among males (OR = 12, p = 0.07) and females (OR = 11, p = 0.24) from DD familes. We found also a slight tendency for higher than expected occurrence of laryngeal cancer among males from DD families (OR = 2.750, p = 0.12). A reverse tendency was observed for non-melanoma skin cancers among females (OR = 0.182, p = 0.68).

For analysis by cancer site, the statistical power *a posteriori* ranged between 0.81 in the most favourable case (male central nervous system tumors) and 0.02 in the least favourable case (several types of cancer, e.g. cervical cancer) (Table 2).

**Table 2:** Expected and observed frequencies, related risk of occurrence of malignancies of different site of origin in Dupuytren’s disease families

**Tumor site**	**Gender**	**Observed**	**Expected**	**Relative risk (RR), p-value**	Statistical power**
		**cases**	**cases**		
Breast	Females	40	33	1.212, p = 0.5080	0,04
Lungs	Males	36	29	1.241, p = 0.4516	0,002
	Females	6	13	0.462, p = 0.1670	0,06
Colorectal	Males	22	28	0.786, p = 0.4756	0,09
	Females	11	15	0.733, p = 0.5542	0,007
Stomach	Males	17	7	2.429, p = 0.651	0,08
	Females	5	4	1.250, p = 1.0000	0,004
Melanoma	Males	2	2	1.000, p = 0.6168	0,002
	Females	1	3	0.333, p = 0.6168	0,02
Kidney	Males	5	6	0.833, p = 1.0000	0,008
	Females	6	4	1.500, p = 0.7513	0,01
Prostate	Males	23	18	1.278, p = 0.5292	0,003
Larynx	Males	11	4	2.750, p = 0.1204	0,05
	Females	0	1	NA	0,05
CNS	Males	13	1	12.00, p = 0.0704*	0,81
	Females	11	1	11.00, p = 0.2392*	0,65
Leukaemia	Males	5	3	1.667, p = 0.7233	0,004
	Females	4	3	1.333, p = 1.0000	0,005
Brain	Males	2	3	0.667, p = 1.0000	0,009
	Females	4	3	1.333, p = 1.0000	0,005
Bones	Males	3	0,3	NA	0,06
	Females	1	0,3	NA	0,008
Bladder	Males	4	10	0.400, p = 0.0953	0,16
	Females	2	3	0.667, p = 1.0000	0,004
NHL	Males	4	2	2.000, p = 0.6828	0,006
	Females	2	2	1.000, p = 0.6168	0,002
Pancreas	Males	4	3	1.333, p = 1.0000	0,003
	Females	7	3	2.333, p = 0.3418	0,05
Uterus	Females	6	11	0.546, p = 0.3303	0,09
Ovary	Females	4	8	0.500, p = 0.3853	0,03
Thyroid	Males	5	2	2.267, p = 0.2202	0,02
	Females	1	4	0.250, p = 0.3701	0,04
Cervix	Females	4	4	1.000, p = 0.4793	0,002
Non-melanoma skin cancer	Males	6	9	0.667, p = 0.6046	0,03
	Females	2	11	0.182, p = 0.6786*	0,33
Esophagus	Males	1	2	0.500, p = 0.9994	0,02
	Females	1	0,5	NA	0,004
Gallbladder	Males	1	0,4	NA	0,004
	Females	0	2	NA	0,14
Liver	Males	2	2	1.000, p = 0.6168	0,003
	Females	2	1	2.000, p = 1.0000	0,007
HL	Males	0	1	NA	0,06
	Females	1	1	1.000, p = 0.4793	0,002
Multiple myeloma	Males	0	1	NA	0,06
	Females	1	1	1.000, p = 0.4793	0,002
Testicle	Males	1	2	0.500, p = 1.0000	0,02

*p-after Bonferroni correction.

** - ≥ 0.7 regarded as statistically powerful.

Molecular examination of 20 mutations/polymorphisms in 12 cancer susceptibility genes in Dupuytren’s patients and controls showed a statistically significant association of one mutation with Dupuytren disease: D312M in *XPD* (OR = 1.75, p = 0.004). There were no other differences in the distribution of cancer susceptibility mutations among DD families and healthy controls (Table 3). The SNP rs2020955 within *XPF* was monomorphic: the frequency of rs2020955_TT among cases was 499 (100%) and among controls 1340 (96,75%).

**Table 3:** Prevalence of the examined mutations/polymorphisms among DD cases and healthy controls

**Gene/Mutation**	** Cases**	** Controls**	** p**	** OR**
BRCA1/5382insC	2/496 = 0,40%	1/496 = 0,19%	0,5346	2,105
BRCA1/4153delA	3/496 = 0,60%	0/496 = 0%		
BRCA1/C61G	0/521 = 0%	1/521 = 0,19%		
CHK2/I157T	30/505 = 5,94%	26/505 = 5,15%	0,5416	1,183
CHK2/CHIVS2	5/505 0,99%	4/512 = 0,78%	0,7222	1,270
NOD2/3020insC	43/505 8,51%	45/493 = 9,13%	0,7328	0,9266
XPD/936 (D312M)	TT 160/504 31,75%	TT 196/484 = 40,49%	**-**	**-**
	CT 193/504 = 38,29%	CT 193/484 = 39,88%	0,6103	0,9357
	CC 151/504 = 29,96%	CC 95/484 = 19,63%	0,004*	**1,752**
XPD/2253 (K751Q)	AA 152/503 = 30,22%	AA 191/524 = 36,45%	**-**	**-**
	AC 268/503 = 53,28%	AC 245/524 = 46,76%	0,732*	1,299
	CC 83/503 = 16,50%	CC 88/524 = 16,79%	0,8998	0,9791
MC1R/R151C	CC 455/500 = 91,00%	CC 453/500 = 90,60%	-	-
	CT 44/500 = 8,80%	CT 45/500 = 9,00%	0,9116	0,9756
	TT 1/500 = 0,20%	TT 2/500 = 0,40%	0,5631	0,4990
MC1R/V60L	GG 427/499 = 85,57%	GG 454/512 = 88,67%	-	-
	GT 68/499 = 13,63%	GT 53/512 = 10,35%	0,1087	1,366
	TT 4/499 = 0,80%	TT 5/512 = 0,98%	0,7672	0,8194
MC1R/R163Q	GG 460/499 = 92,18%	GG 463/503 = 92,05%	-	-
	AG 39/499 = 7,82%	AG 37/503 = 7,35%	0,7834	1,068
	AA 0/499 = 0%	AA 3/503 = 0,60%	0,0840	0,1431
VDR/M1T	GG 135/504 = 26,79%	GG 161/518 = 31,08%	-	-
	AG 277/504 = 54,96%	AG 262/518 = 50,58%	0,1607	1,192
	AA 92/504 = 18,25%	AA 95/518 = 18,34%	0,9717	0,9943
CDKN2A/A148T	25/504 = 4,96%	23/490 = 4,69%	0,8447	1,060
p53/P72R	GG 260/496 = 52,42%	GG 269/513 = 52,44%	-	-
	CG 188/496 = 37,90%	CG 210/513 = 40,93%	0,3245	0,8807
	CC 48/496 = 9,68%	CC 34/513 = 6,63%	0,0763	1,509
rs6983267	GG 122/500 = 24,40%	GG 135/519 = 26,01%	-	-
	GT 262/500 = 52,40%	GT 260/519 = 50,10%	0,4621	1,097
	TT 116/500 = 23,20%	TT 124/519 = 23,89%	0,7946	0,9623
ATM/E1978X	1/505 = 0,20%	0/505 = 0%	NA	NA
MTHFR/(A222V	CC 191/415 = 46,02%	CC 229/500 = 45,80%	-	-
	CT 185/415 = 44,58%	CT 227/500 = 45,40%	0,8036	0,9673
	TT 39/415 = 9,40%	TT 44/500 = 8,80%	0,7540	1,075
XPC/A499V	GG 299/489 = 61,15%	CC 258/489 = 52,7%	**-**	**-**
	AG 167/489 = 34,15%	CT 194/489 = 39,6%	**0**,2083	0,8679
	AA 23/489 = 4,70%	TT 33/489 = 6,70%	0,3245	0,7098
XPD/rs238406 (c.396A > C)	CC 173/492 = 35,16%	CC 141/492 = 28,66%	**-**	**-**
	AC 230/492 = 46,75%	AC 267/492 = 54,27%	0,098*	0,7407
	AA 89/492 = 18,09%	AA 84/492 = 17,07%	0,5994	1,076
XPF/rs762521 (c.207 + 11G > A)	GG 266/478 = 55,65%	GG 280/478 = 58,57%	-	-
	AG 182/478 = 38,07%	AG 167/478 = 34,94%	0,2083	1,150
	AA 30/478 = 6,28%	AA 31/478 = 6,49%	0,8696	0,9644

*p-value after Bonferroni correction.

For the analysis of genetic markers, the statistical power *a posteriori* ranged between 0.12 in the most favourable case (marker rs6983267, variant GT) and 0.001 in the least favourable case (marker *CHK2*, variant IVS2 + 1G > A). Comparatively, for the most favourable case, the minimum sample size for a statistical power *a priori* of at least 0.7 should have been greater than 1800 cases and an equivalent number of controls.

## Discussion

Assessment of the risk of cancer as a single outcome revealed no statistically significant association between DD and disease. It indicates that the most common malignancies, such as breast, prostate, lung or colon cancers are not strongly associated Dupuytren disease. However, it does not exclude a moderate association for rarer malignancies. Evaluation of the tumor spectrum of cancers and comparison of the observed and expected frequencies revealed a tendency towards over-representation of central nervous system tumors and laryngeal cancer among male patients in DD families. We also found a tendency towards under-representation of non-melanoma skin cancers between female relatives of DD cases. Due to the relatively small number of cases, both type 1 and 2 statistical errors cannot be excluded; the results thus need to be verified by examination of a larger series of patients. The current study lacks the statistical power to confirm if the absence of any observed association is due to a true lack of association or rather due to insufficient sample size. The current research should be considered only as an exploratory study attempting to reveal interesting tendencies worthy of in-depth analysis at a later stage.

There are only few reports in the literature regarding the association of Dupuytren’s disease with malignant neoplasms. The only available studies were based on the statistical analysis of basic population-characteristic variables, such as morbidity and mortality rates. Gudmundsson et al. [1], reported increased total mortality and cancer mortality in men with advanced Dupuytren’s disease in an Iceland population (hazard ratio 2.0; 95% CI 1.0-3.7). The incidence of cancer was moderately, but not statistically significantly higher than expected from the reference cohort. There was also an insignificant predominance of digestive tract cancers among DD patients [1].

Wilbrand et al. [11] determined the risk of cancer in 15 212 patients operated on for DD, by means of record linkage to the Swedish Cancer Register. The overall relative risk of cancer in the DD patient population was increased by 24%, compared to the general population. There were significantly increased risks of malignancies related to smoking, such as buccal, oesophageal, laryngeal, gastric, lung and pancreatic cancers. Significantly increased risks were present for both prostate and rectal cancer in men, and an increased risk for breast cancer in women was noted one year or more after surgery for DD. Based on their findings, the authors suggest that DD patients display other characteristics, which alter the risk of malignacies when compared with the general population [11]. In their later study, these authors reported an increased frequency of soft tissue sarcomas: fibrosarcoma, malignant fibrous histiocytoma and chondrosarcoma among DD patients. These authors noted that neither smoking, diabetes, nor cancer syndromes could explain why patients with DD have a higher incidence of sarcoma [3].

Unfortunately, the results of our investigations are not consistent with those reported above, as we did not find any significant association between Dupuytren’s disease and malignant neoplasms.

Examination of mutations/polymorphisms of cancer susceptibility genes revealed significant association of the D312M variant of *XPD* with Dupuytren disease (OR = 1.75; p = 0,004). The variant is has been reported to predispose to melanoma, cancers of the lung, breast, liver and stomach [12-19]. We observed no over-representation of these malignancies in DD families. This could be explained by low statistical power of our study and/or the fact that D312M is associated only with low-to moderate cancer risk.

The results of molecular genotyping of the remaining mutations/polymorphisms present in cancer susceptibility genes revealed no differences in the prevalence of the examined alterations between cases and controls. It seems that none of the examined alterations in DNA are associated with Dupuytren disease.

Smoking is well documented risk factor for the development of many malignant neoplasms, including lung, laryngeal, buccal, oesophageal and pancreatic cancers. It is also known to be a risk factor for Dupuytren’s disease. In our study we failed to find any significant association between Dupuytren’s disease and malignant neoplasms, including those related to smoking habit. We believe that this potential link need to be further investigated for better understanding of the underlying biological mechanisms leading to both diseases.

In conclusion, the results of our study indicate a lack of a strong association between Dupuytren disease and familial cancer risk. We observed only one significant association of the D312M variant of the XPD gene with DD and a tendency toward changed frequencies of occurrence of central nervous system tumors, laryngeal cancer and non-melanoma skin cancers in DD families. Since Dupuytren contracture is polygenetic, it would be also interesting to know whether there are interactive effects between the examined SNPs. As a future option, large sample sizes are needed to investigate such genetic interactive effects. Further studies are needed to confirm the findings and to evaluate whether or not cancer surveillance protocols are justified in these families.

## Competing interest

The authors declare that they have no competing interest.

## Authors’ contribution

KPS carried out molecular genotyping; SG performed statistical analyses; AZ, RS. JL and TD participated in the sequence alignment and drafted the manuscript. All authors read and approved the final manuscript.
